# siRNA-mediated inactivation of HER3 improves the antitumour activity and sensitivity of gefitinib in gastric cancer cells

**DOI:** 10.18632/oncotarget.17526

**Published:** 2017-04-29

**Authors:** Heng-Heng Yuan, Ying-Nan Yang, Jian-Hua Zhou, Yan-Jing Li, Li-Ying Wang, Jun-Wei Qin, Tao Liu, Zhen-Zhen Li, Qing-Xin Zhou, Xiao-Li Wei, Ting-Ting Zhang, Peng Huang, Wen-Jie Zhang, Lei Liu, Xiao-Xue Du, Yu Han

**Affiliations:** ^1^ Department of Gastrointestinal Oncology, Harbin Medical University Cancer Hospital, Harbin, Heilongjiang Province, China; ^2^ Department of Chest Surgery, Harbin Medical University Cancer Hospital, Harbin, Heilongjiang Province, China; ^3^ Department of Oncology, Chaoyang Central Hospital, Shenyang, Liaoning Province, China; ^4^ Department of Oncology, Qingdao Municipal Hospital, Qingdao, Shandong Province, China; ^5^ The Third Department of Oncology, Xinxiang Central Hospital, Xinxiang, Henan Province, China

**Keywords:** HER3, siRNA, gefitinib, gastric cancer, PI3K/AKT

## Abstract

The human EGFR family consists of four type-1 transmembrane tyrosine kinase receptors: HER1 (EGFR, ErbB1), HER2 (Neu, ErbB2), HER3 (ErbB3), and HER4 (ErbB4). HER3 can dimerize with EGFR, HER2 and even c-Met and likely plays a central role in the response to EGFR-targeted therapy. Because HER3 lacks significant kinase activity and cannot be inhibited by tyrosine kinase inhibitors, neutralizing antibodies and alternative inhibitors of HER3 have been sought as cancer therapeutics. Here, we describe the stable suppression of HER3 mRNA and protein using siRNA. The inhibition of HER3 expression decreased cell proliferation, suppressed cell cycle progression, induced apoptosis and inhibited cell motility, migration, invasiveness, and soft agar growth. In addition, we found that gefitinib treatment increased the HER3 and HER2 mRNA levels. The administration of various concentrations of gefitinib to HER3-knockdown cells enhanced antitumour activity and sensitivity due to the downregulation of protein phosphorylation via PI3K/AKT and ERK signalling. Our results support the use of combined treatments targeting multiple EGFR receptors, particularly the use of HER3 inhibitors combined with EGFR inhibitors, such as gefitinib.

## INTRODUCTION

Gastric cancer (GC) is one of the most frequently diagnosed cancers and the second leading cause of cancer-related mortality worldwide [1]. Despite recent progress in early diagnosis and improvements in therapeutic regimens, many patients still develop advanced disease and have poor clinical outcomes. The median overall survival (OS) is 8–10 months, and the five-year survival for metastatic GC is less than 7% [2]. Because optimal combinations of surgery and chemotherapy have not achieved the expected survival outcomes, the pivotal role of targeted therapeutics has received increasing attention.

The human epidermal growth factor receptor (EGFR/HER) family is involved in multiple complex and tightly controlled signalling pathways that regulate various cellular functions, including cell proliferation, organ development, and organ repair [3–5]. Aberrant HER signalling has been associated with the development of various types of solid tumour [6]. The four members of the HER family, HER1 (EGFR), HER2, HER3 and HER4, share a common extracellular ligand-binding domain, a transmembrane domain and an intracytoplasmic tyrosine domain [7, 8]. Of the HER family members, EGFR and HER2 are the most well-documented proto-oncogenes. At least nine cancer therapeutics [monoclonal antibodies (mAbs) and small-molecule tyrosine kinase inhibitors (TKIs)] targeting EGFR and/or HER2 are currently in clinical use [3, 9–13]. Although their clinical benefits have been demonstrated, these drugs induce different patient responses, and drug resistance is often encountered [14]. The mechanisms resulting in refractory and acquired resistance to anti-HER agents are poorly understood. Different models, including genetic mutations of key genes in the HER pathway, such as KRAS and PTEN, and upregulation of oncogenes, such as cMET and HER3, have been proposed [15–17].

Data acquired over the past few years have revealed that HER3 plays a vital role in cancer growth and progression [18]. In contrast to HER1, HER2, and HER4, all of which possess active tyrosine kinase domains, HER3 lacks intrinsic kinase activity, and a HER3 homodimer has not been reported. It is now clear that heregulin binding to HER3 or HER3 dimerization with HER1 or HER2 leads to hyperphosphorylation of the cytoplasmic tail of HER3, which in turn drives AKT signalling [19–21]. HER3 plays a key role in maintaining the equilibrium of HER family member dimerization and signalling and in sensing perturbations in these processes, and emerging evidence from both laboratory and clinical observations strongly validates HER3 as a cancer drug target [15, 16]. Experimental downmodulation of HER3 using genetic or pharmacologic methods supports the notion that HER3 is an important driver of cancer growth. Consistent with this finding, the targeting of HER3 by conditional knockout or using LNA-based antisense molecules inhibits lung adenocarcinoma tumour growth [22] as well as ceases disease progression and prolongs survival in xenograft mouse models of ovarian cancer [20] or HER2-overexpressing breast cancer [23]. Furthermore, the *in vitro* targeting of HER3 using siRNAs and antisense deoxyoligonucleotides (AS-ODNs) potently inhibits cellular proliferation and promotes apoptosis in cells sensitive and insensitive to HER1 and HER2 inhibitors [20, 22–25].

In the present study, we further demonstrate the importance of HER3 in gastric cell malignancy using RNA interference, a powerful tool. Specifically, we show that the siRNA-directed downmodulation of HER3 inhibits GC cell proliferation, motility, invasion and survival and promotes apoptosis. Furthermore, the evidence obtained in the present study suggests that the combination of HER3 siRNA with gefitinib has greater efficacy than gefitinib alone and might overcome insensitivity to TKIs, such as gefitinib.

## RESULTS

### HER3 siRNA decreases the proliferation and increases the apoptosis of MKN45 cells

**Figure 1:**
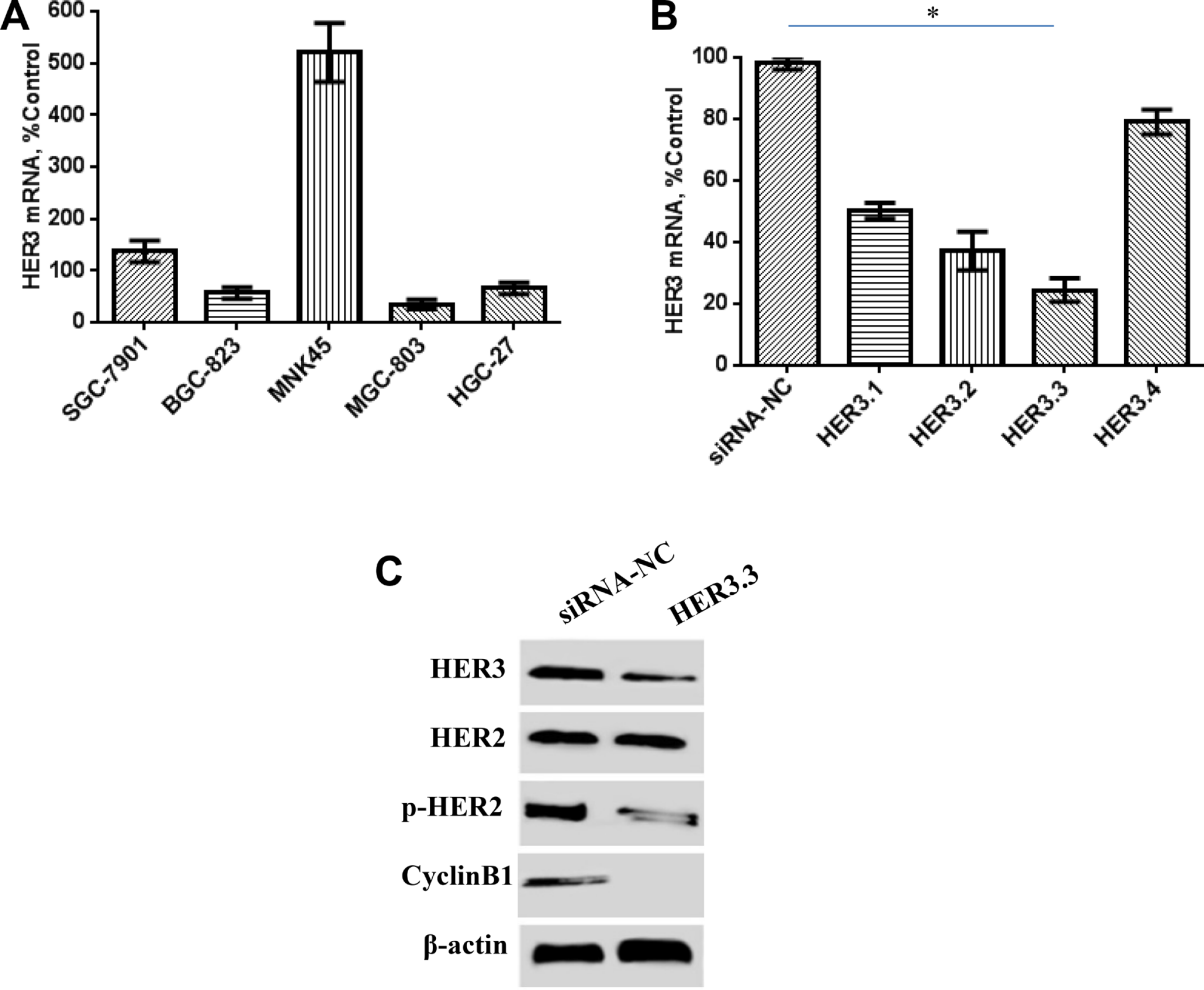
Effect of HER3 siRNA on the HER3 mRNA and protein levels (**A**) HER3 mRNA expression is relatively high in human MKN45 GC cells, as detected by real-time PCR. (**B**) The HER3.3 siRNA construct is more effective than the other siRNA constructs. A non-silencing siRNA construct (control NS) was used as a control. The data represent the HER3 mRNA expression levels relative to that of GAPDH and are presented as the means ± SDs; *P < 0.05. (**C**) HER3.3 downmodulates HER3 protein expression in MKN45 cells. The knockdown of the HER3 level reduced the levels of activated HER2 (p-HER2), but not total HER2. HER3 knockdown in MKN45-HER3.3 cells also decreased Cyclin B1 expression.

The HER3 mRNA levels in five GC cell lines were investigated through real-time RT-PCR. MKN45 cells showed relatively higher HER3 expression than the other four GC cell lines (Figure 1A). Synthetic siRNAs directed against different regions of HER3 (HER3.1, HER3.2, HER3.3 and HER3.4) were then tested for their ability to suppress HER3 mRNA expression, and the results showed that HER3.3 siRNA was more effective than the others. An approximate 80% reduction in mRNA levels was observed in the cells transduced with HER3.3 compared with the control cells (Figure 1B). To ensure that the reduction in mRNA was associated with a reduction of protein expression, we conducted a Western blotting analysis of cellular lysates, and the results showed that HER3.3 downmodulated the HER3 protein levels (Figure 1C). Because HER2 is the other important component of the HER2/HER3 heterodimer, the expression and activity of HER2 in the transduced cells were also detected. Interestingly, decreases in the HER3 level decreased also reduced the levels of activated HER2 (p-HER2), but not total HER2, compared with the control cells (Figure 1C).

**Figure 2:**
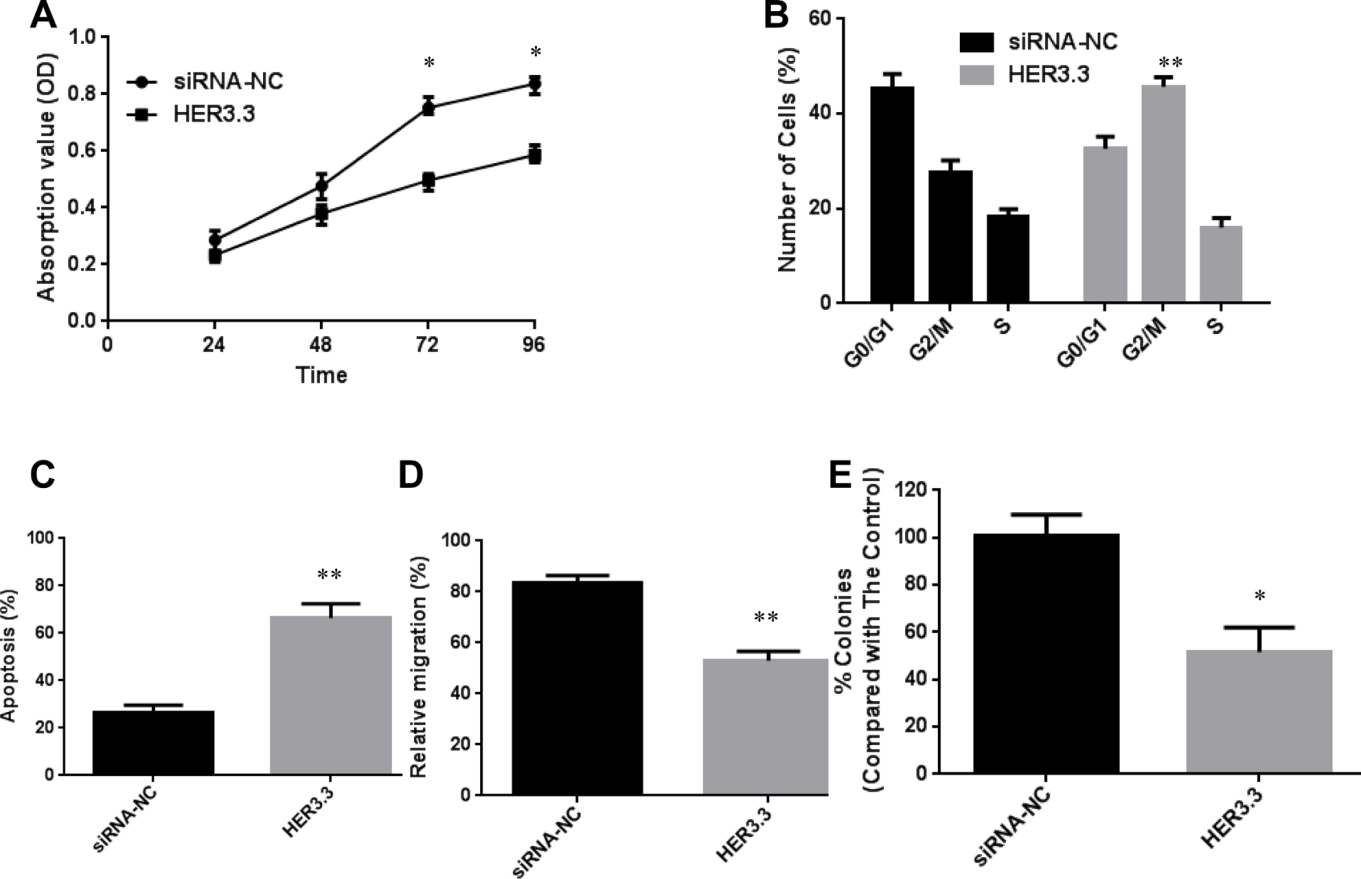
Downregulation of HER3 decreases cell proliferation and increases apoptosis (**A**) Cell growth curves for MKN45-HER3-cs and MKN45-HER3.3 cells based on MTT assay results. (**B**) Comparison of cell cycle distributions between different groups. (**C**) TUNEL assay-based analysis of apoptosis in the indicated groups. (**D**) Comparison of the relative migration abilities between the indicated groups. E. Inhibition of MKN45 cell growth in soft agar by HER3.3. All of the data are shown as the means ± SDs from three experimental replications; *P < 0.05; **P < 0.01.

As mentioned above, HER3 plays a vital role in cancer growth and progression. A series of *in vitro* experiments was designed to explore the mechanism underlying this behaviour in detail. First, an MTT assay was performed to investigate the effect of HER3 downregulation on the proliferation of MKN45 cells, and the results showed that HER3 downregulation significantly decreased cell proliferation (Figure 2A). Furthermore, a cell cycle analysis revealed that the blockage of HER3 caused G2/M arrest: the percentage of MKN45-HER3.3 cells in the G2/M phase was 45.4 ± 4.2%, whereas only 23.8 ± 2.4% of MKN45-HER3-cs cells were found in this phase (*P* = 0.0061, Figure 2B). Because G2/M arrest was observed after the knockdown of HER3, the expression of Cyclin B1, which regulates the transition from the G2 to the M phase, was investigated. Decreased Cyclin B1 expression was detected, and this finding helps explain why HER3 knockdown in MKN45-HER3.3 cells causes G2/M arrest (Figure 1C). An *in vitro* apoptosis assay showed that the blockage of HER3 expression in MKN45-HER3.3 cells substantially increased the apoptotic rate to 67.2 ± 8.37% compared with the apoptotic rate of the control cells (26.3 ± 5.08%), as assessed by a TUNEL assay (Figure 2C).

### HER3 siRNA inhibits MKN45 cell migration and growth in soft agar

MKN45 cells have high migration potential and were therefore used in our cellular migration assays. Their migration ability was assessed using a wound-healing assay, and the results showed that the relative migration rate decreased from 81.3 ± 6.4% in MKN45-HER3-cs cells to 57.4 ± 5.2% in MKN45-HER3.3 cells (Figure 2D). We next investigated the effect of HER3 downregulation on anchorage-independent growth by assaying colony formation on soft agar, which is an indicator of malignancy. HER3.3 caused an approximate 50% reduction in the number of soft agar colonies compared with that observed with the control cells (Figure 2E), confirming the importance of HER3 in tumour growth and survival.

### HER3 siRNA potentiates the antiproliferative effect of gefitinib

Gefitinib (Iressa) is a TKI that specifically targets EGFR and has been used to treat non-small cell lung cancers with EGFR mutations. However, only approximately 10–15% of patients are sensitive to this drug [26]. Some reports support the use of combined treatments targeting multiple EGFRs; for example, the combination of the TKI with the neutralizing HER3 antibody AMG-888 shows better efficacy than either agent alone [27], and the combination of the PI3K inhibitor BEZ235 with trastuzumab also augments the antitumour effect of either drug alone [28]. EGFR gene mutation and compensatory signalling from HER3 and other EGFR family members have been suggested to be associated with sensitivity to gefitinib, which could ultimately result in resistance to the drug [29]. Therefore, we hypothesize that the treatment of cancer cells with the combination of HER3 siRNA and gefitinib would improve the treatment efficacy and induce a more sensitive response than gefitinib alone.

**Figure 3:**
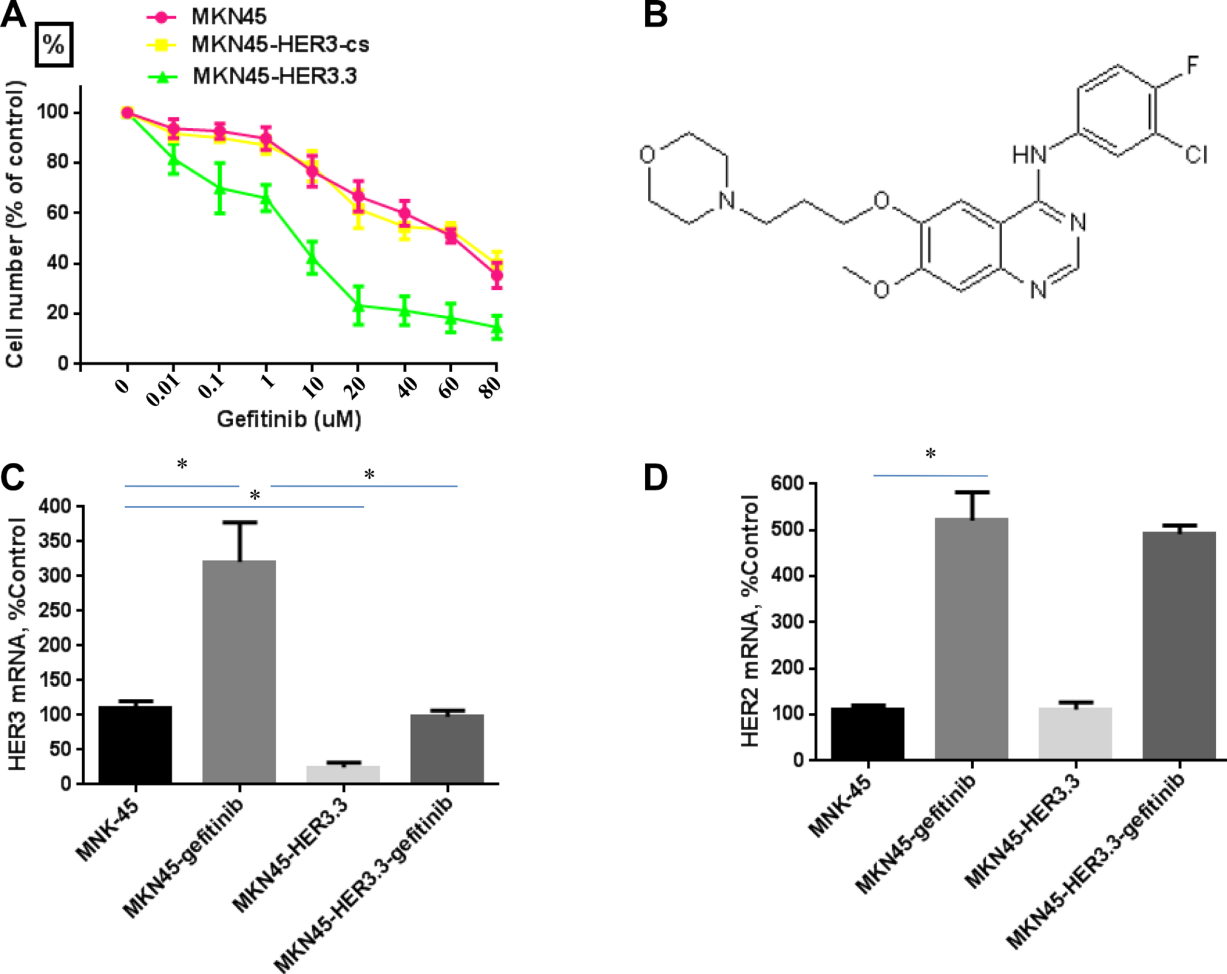
HER3 siRNA potentiates the effect of gefitinib (**A**) Comparative growth inhibition rates of MKN45, MKN45-HER3-cs and MKN45-HER3.3 cells exposed to gefitinib. Control siRNA- and HER3.3-transfected MKN45 cells (2 × 10^3^ cells/well) were grown in DMEM supplemented with 10% FBS in 96-well plates for 48 h and treated with various concentrations of gefitinib for 48 h. HER3.3 potentiates the effect of gefitinib on MKN45 cell growth, as determined by an MTT assay. The results represent the means ± SDs of three independent experiments. (**B**) Structure of gefitinib. Gefitinib treatment of MKN45 cells significantly induces HER3 (**C**) and HER2 (**D**) mRNA expression, and this induction is prevented by HER3.3 (C). Induction of HER2 expression by gefitinib is not prevented by HER3.3 (D). *P < 0.05 compared with control cells.

The sensitivity and IC50 for gefitinib were determined using MTT assays of MKN45, MKN45-HER3-cs and MKN45-HER3.3 cells after treatment with various concentrations of gefitinib for 48 h. The MKN45 and MKN45-HER3-cs cell lines were resistant to gefitinib and had IC50 values greater than 40 μM (Figure 3A). The structure of gefitinib is shown in Figure 3B. A previous study showed that treatment with gefitinib increases the HER3 mRNA levels [29]. Consistent with this finding, gefitinib (20 μM) significantly increased the HER3 mRNA expression level in MKN45 cells by more than three-fold (Figure 3C), and an approximate five-fold induction of HER2 mRNA (Figure 3D) was also found. Interestingly, the growth of the MKN45-HER3.3 cell line was markedly inhibited by gefitinib, with an IC50 of 9.8 ± 3.34 μM (Figure 3A). These results indicate that the combined treatment enhanced the antitumour activity of and sensitivity to gefitinib. Finally, the gefitinib-mediated induction of HER3 expression appeared to be prevented by HER3.3 (Figure 3C).

### Combination treatment with gefitinib and HER3 siRNA inhibits the PI3K/AKT and ERK signalling pathways

**Figure 4:**
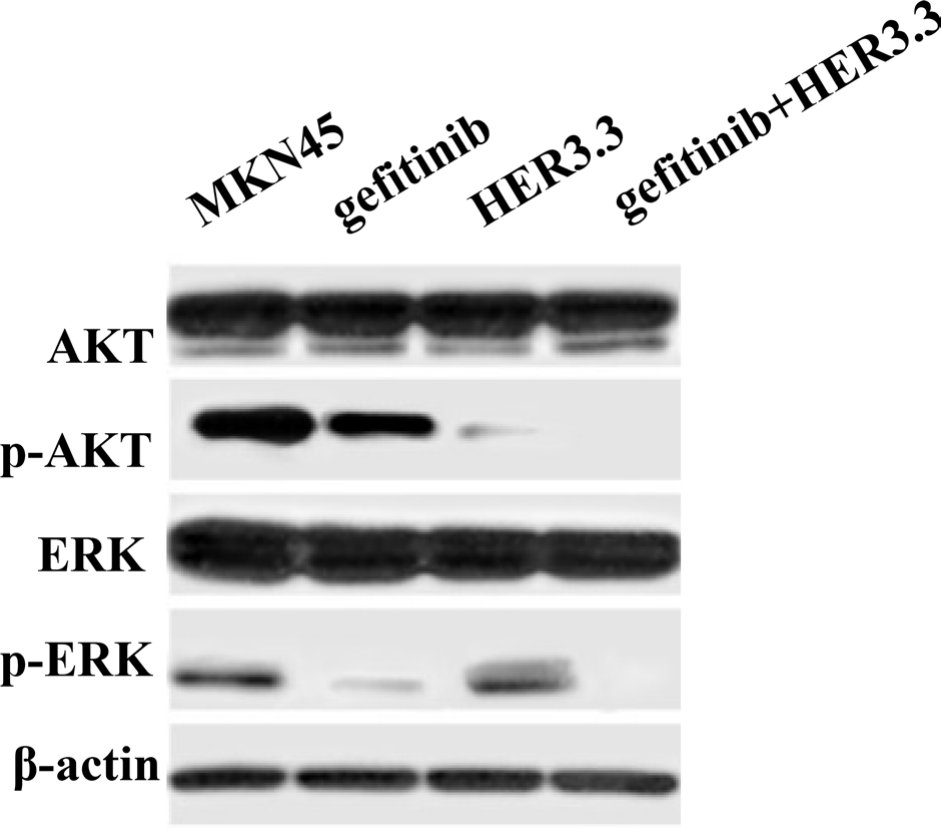
Combination of gefitinib and HER3 siRNA inhibits the PI3K/AKT and ERK signalling pathways MKN45 and MKN45-HER3.3 cells were treated with 20 μM gefitinib. After 48 h of treatment, AKT and ERK activation is simultaneously ablated by the combination compared with the single treatments and the control. HER3.3 yields a greater inhibition of p-AKT than gefitinib.

To identify the mechanism underlying the enhanced sensitivity to gefitinib following HER3 downregulation in MKN45 cells, a Western blotting assay was performed to assess alterations in two EGFR-related signalling pathways, the PI3K/AKT and ERK pathways, following gefitinib treatment. As shown in Figure 4, the combination of gefitinib and HER3.3 more potently inhibited both pathways compared with either agent alone. The inhibition of HER3 by HER3.3 affected mainly the p-AKT level, whereas only moderate ERK alteration was observed.

## DISCUSSION

HER3 has been implicated in oncogenic proliferation in various cancer types [30], such as HER2-overexpressing breast cancer cells and ovarian cells. The results obtained in this study provide substantial evidence supporting the hypotheses that HER3 plays a role in GC cell proliferation, survival and invasion and that these effects are mediated through PI3K/AKT signalling, which is important for cell survival and dysregulated in most cancers. The data are consistent with the function of HER3 in cancer cells. Upon binding to HRG1, the physiological HER3 receptor ligand, HER3 dimerizes with other ErbB family members, with a preference for HER2. This dimerization results in the transphosphorylation of HER3 on tyrosine residues contained within the cytoplasmic tail of the protein [31–33]. The phosphorylation of these sites creates SH2 docking sites for SH2-containing proteins, specifically PI3-kinase [34]. HER3 is a potent activator of AKT because it possesses six tyrosine phosphorylation sites with YXXM motifs, which serve as excellent binding sites for the p85 regulatory subunit of PI3K, resulting in activation of the downstream AKT pathway [35, 36]. These six PI3K sites strongly amplify HER3 signalling, and activation of the pathway further elicits several important biological processes, including cell growth and survival [37]. Consistent with these relationships, we observed that reductions in HER3 expression decreased the activity of AKT and HER2 but did significantly change HER2 expression.

HER3 has not been the focus of many studies due to its impaired kinase activity. However, recent basic and clinical evidence has indicated that HER3 plays an important role in HER signalling and drug resistance [38]. For example, HER3 has been shown to mediate resistance to the EGFR inhibitor gefitinib in both breast [37] and lung cancer cells [39]. Thus, HER3 appears to be a critical primary target for the development of cancer therapies, perhaps in conjunction with EGFR, HER2 and/or AKT targeting and/or chemotherapy. Unlike HER1 and HER2, HER3 does not appear to have appreciable kinase activity; therefore, ATP-mimetic small-molecule inhibitors might not be able to inhibit HER3-mediated tumour growth. However, inhibition of HER3 activity can also be achieved using antibodies that block ligand binding to HER3, although the possibility that the cytoplasmic tail of HER3 can become phosphorylated and thereby hyper-activated by other growth factors [19] suggests that these antibodies might not be effective in all patients. Resistance to these mAb agents is also highly likely because the phosphorylated tail of HER3 still engages the critical PI3K pathway. In addition, isoforms of HER2 that are devoid of the extracellular domain are expressed in many breast cancers [15] and might thus prohibit the use of antibodies that target HER2 or HER2/3 heterodimers. Therefore, an RNA antagonist to HER3 offers a unique solution for controlling HER3-mediated tumour growth. The results of this study show that HER3 siRNA can be used to specifically inhibit HER3 through the downmodulation of HER3 mRNA and protein and, consequently, the p-AKT levels. siRNA reagents were demonstrated to be effective experimental tools in this study and could be useful for future therapeutic approaches.

The hyperactivation of EGFR is known to mediate tumour growth, and consistent with this finding, small molecules and antibodies that bind to HER1, such as gefitinib, have proven anti-cancer activity. However, the results from an international Phase II randomized clinical trial consisting of 75 stage IV gastric cancer patients were not as hopeful as expected: many patients showed no response at all (0%), some had a very low partial response rate (1.4%), and a minority exhibited stable disease (17%). Overall, the therapeutic efficacy of gefitinib was quite low (18.3%) [40, 41]. The compensatory overexpression of other HER family members has been implicated as one of the mechanisms driving this resistance to gefitinib, and the inhibition of HER3 signalling appears to be important for gefitinib-induced apoptosis in gefitinib-sensitive lung cancer cells [40]. In addition, hyperactivation of HER3 has also been reported to negatively correlate with the response of lung cancer cells to anti-EGFR therapy [15]. Sergina et al. showed that long-term treatment of SKBR3 cells with gefitinib causes re-localization of HER3 from intracellular compartments to the plasma membrane, resulting in increased levels of activated HER3 and reactivation of the AKT pathway [37]. Based on these relationships, the results reported by Grøvdal et al. support the use of combined treatments targeting multiple ErbB receptors, particularly HER3 inhibitors combined with EGFR inhibitors, such as gefitinib [29]. Another logical option is to consider the combination of a HER3 antagonist, such as HER3 siRNA, and gefitinib.

In this study, we showed that gefitinib treatment leads to upregulation of the HER3 mRNA levels. Interestingly, gefitinib did not appear to induce HER2 expression but did increase the HER2 mRNA levels, effects that could have resulted from a feedback mechanism that compensated for the inhibition of ErbB receptor signalling and have been linked to the downregulation of HER2/HER3 activity and the AKT pathway via FoxO3a [27]. Similar to our results for HER2, HER3 and EGFR have been reported to be upregulated in breast cancer cells after long-term treatment with the HER2-targeting antibody trastuzumab [42]. Thus, cancer cells might frequently upregulate other ErbB receptors as a compensatory mechanism when signalling from one receptor is inhibited.

We reasoned that treatment of HER3-downregulated cancer cells with gefitinib would improve the efficacy of and sensitivity to the drug. Indeed, the combination of both treatments resulted in greater antitumour activity and higher sensitivity compared with those observed in cells that were exposed to gefitinib alone. Our data are in agreement with a report by Schaefer and colleagues, who showed the benefit of combining agents to target multiple family members using a two-in-one antibody against HER3 and EGFR [43]. It is worth noting that the neutralizing HER3 antibody AMG has been shown to sensitize HER-positive BT474 breast cancer cells to lapatinib [27]. In our case, the improvement in the efficacy of gefitinib is clearly an on-target effect because the treatment of MKN45 cells with HER3.3 suppressed the three-fold induction in HER3 mRNA expression that was caused by gefitinib. The combination of HER3.3 with TKIs or inhibitors of the PI3K/AKT pathway might thus provide a novel and highly effective approach for the treatment of various cancers.

The most important finding of this study is the demonstration of a possible mechanism associated with, as well as the biological significance of, the PI3K/AKT and ERK pathways in gefitinib-treated gastric cancer cell lines with or without downmodulation of HER3. Previous studies have shown that HER3 activity plays an important role in AKT activation [32, 44] and is induced by gefitinib, possibly due to the inhibition of AKT activity, which in turn induces HER3 expression through FOXO [45]. The inhibitory effect of gefitinib on AKT activation is, however, reduced because elevated HER3 levels activate AKT, preventing its complete inhibition. Our data demonstrate that the combination of gefitinib and HER3.3 as well as HER3.3 alone more potently reduces AKT activity and exerts an increased inhibitory effect on cell growth than gefitinib alone. More importantly, the knockdown of HER3 results in a reduction of activated HER2, suggesting that HER2/HER3 heterodimerization contributes to gefitinib resistance. This notion is consistent with the results of previous studies, which demonstrated that increased HER2/HER3 dimerization leads to the activation of both HER2 and HER3 as well as their downstream signalling pathway in cancer [46, 47].

Collectively, these data suggest that the siRNA-mediated downmodulation of HER3 offers a unique opportunity to confirm HER3-driven cancers. HER3 expression is upregulated after gefitinib treatment, suggesting that HER3 might be the key to gefitinib resistance. In addition, our results provide the first demonstration that the dual targeting of EGFR and HER3 in GC cells is more effective than the targeting EGFR alone. The development of new biomarker strategies is necessary to increase the success rates of clinical trials and maximize the benefits to patients. Some preclinical data suggest that expression of the ligand neuregulin-1 is associated with HER3 activation in ovarian [20] and head and neck cancer [21]. Additional preclinical and clinical data as well as data integration will help identify a predictive biomarker for HER3-targeted therapy, and greater clinical benefits might be attained by combining HER3-targeted therapy with other cancer-targeting antibodies and/or small-molecule TKIs.

## MATERIALS AND METHODS

### Cell lines and culture conditions

Cell culture reagents were purchased from Invitrogen unless otherwise mentioned. The human GC cell lines SGC-7901, BGC-823, MNK45, MGC-803 and HGC-27 (GeneChem, Shanghai, China) were grown in RPMI-1640 or Dulbecco’s’ Modified Eagle's Media (DMEM) medium supplemented with 10% heated inactivated foetal bovine serum (FBS), penicillin (100 U/ml) and streptomycin (100 μg/ml) and maintained in a humidified atmosphere with 5% CO_2_ and 95% air at 37°C.

### Reagents and antibodies

Gefitinib (Iressa, TOCRIS, UK) was dissolved in dimethyl sulfoxide (DMSO, Sigma) prior to the experiments. 3-(4,5-Dimethylthiazol-2-yl)-2,5-diphenyltetrazolium bromide (MTT, Amresco, USA) and propidium iodide (PI) were dissolved in PBS to final concentrations of 5 mg/ml and 50 μg/ml, respectively. The Annexin V-APC apoptosis detection kit was obtained from BD Biosciences (BD, NY, USA). Antibodies against HER3, p-HER3, HER2, p-HER2, AKT, p-AKT, ERK, p-ERK and Cyclin B1 were purchased from Cell Signalling Technology (Beverly, MA, USA), and the antibody against β-actin was obtained from Santa Cruz Biotechnology (Santa Cruz, CA, USA).

### siRNA design and lentivirus production and transduction

siRNAs targeting HER3 and a control siRNA were designed and synthesized by GeneChem (Shanghai, China) and subcloned into a lentiviral vector (pGCSIL-GFP) to obtain pGCSIL-GFP-cs and pGCSIL-GFP-HER3-siRNA. The targeted sequences (sense strand) were the following: HER3.1, GTGAGGTGGTGATGGGGAA; HER3.2, GTGGATTCG AGAAGTGACA; HER3.3, CCATCTTCGTCATGTT GAA; and HER3.4, AGACACTGTACAAGCTCTA. Recombinant lentivirus was generated from 293T cells, and MKN45 cells were transduced with lentivirus using polybrene (8 lg/ml).

### Quantitative real-time reverse transcription-polymerase chain reaction (RT-PCR)

Total RNA was isolated using the TRIzol^®^ Reagent (Invitrogen), according to the manufacturer's recommended protocol, and 1 μg of RNA was reversely transcribed using the SuperScript First-Strand Synthesis System (Invitrogen, CA, USA). The RNA templates were treated with DNase I to avoid genomic DNA contamination. Real-time PCR analyses were performed using an Applied Biosystems 7300 Detection System (Applied Biosystems, CA, USA) using primers for HER3 (forward: 5′-CCTGGGACTCTGAATGGC-3′, reverse: 5′-AGCCTGTCACTTCTCGAATC-3′), HER2 (forward: 5′-AGCCCTGGTCACCTACAACA-3′, reverse: 5′-GCA CTGGTAACTGCCCTCAC-3′), GAPDH (forward: 5′-TG ACTTCAACAGCGACACCCA-3′, reverse: 5′-CACCC TGTTGCTGTAGCCAAA-3′). Real-time PCR was performed using the SYBR^@^ Premix Ex Taq^TM^ kit according to the manufacturer's instructions (Takara, Japan). The data were normalized to the GAPDH levels in the samples and calculated using the ΔΔCt method.

### MTT assay

The cultured cells were plated at a density of 5 × 10^3^ cells/well on a 96-well plate. The viability of the cells at 24, 48, 72 and 96 h was evaluated through an MTT assay. MTT was added to each well at a concentration of 500 μg/ml, and the plates were incubated for 4 h at 37°C. After the media were aspirated, the cells were lysed with 400 μl of DMSO. The cells were then incubated for 10 min at 37°C with gentle shaking. The absorbance readings at 570 nM were determined using a computer-controlled microplate analyser.

### Terminal deoxynucleotidyl transferase dUTP nick end labelling (TUNEL) assay

The cells were cultured in medium with 10% FBS for 24 h. A TUNEL assay was performed using an *In Situ* Cell Death Detection Kit (Roche Diagnostics, IN, USA) and strictly following the procedure provided by the manufacturer. The slides were mounted in mounting solution and analysed by fluorescence microscopy (Axiovert 200; Zeiss, Stuttgart, Germany).

### Cell cycle analysis

Cells cultured in complete medium with 10% FBS for 48 h were harvested, washed twice with ice-cold PBS, and fixed in ice-cold 70% (v/v) ethanol overnight at 4°C. Prior to analysis, the ethanol was removed by centrifugation, and the samples were washed again with PBS. The cells were then re-suspended in PBS containing PI (50 μg/ml) and RNase A (10 μg/ml) and incubated at 37°C in the dark for 30 min. The percentages of cells in the G0/G1, S, and G2/M phases were determined.

### Wound-healing assay

The cells were grown in medium supplemented with 10% FBS. The cells were seeded into six-well tissue culture plates at a density of 40 × 10^5^ cells per well. A new 200-μl pipette tip was used to gently and slowly scratch the monolayer at the centre of each well. The resulting gap distance was equal to the outer diameter of the end of the tip. After scratching, the well was washed twice with medium to remove the detached cells and replenished with fresh medium. Following 24 and 48 h of growth, the cells were washed twice with 1× PBS, and the stained monolayer was observed under a microscope to quantitatively evaluate the gap distance using Image-Pro Plus software.

### Colony formation assay

The cells were plated in six-well culture plates at a concentration of 200 cells per well and allowed to form colonies for 14 days. The medium was changed every three days. The colonies were then stained with 0.2% crystal violet with buffered formalin (Sigma). The colony numbers were then manually counted under a microscope, and a colony was defined as a group of at least 50 cells.

### Western blotting analysis

The proteins extracted from GC cell lines were quantified using a protein assay (Bio-Rad Laboratories, CA, USA). The protein samples (30–50 μg) were fractionated by SDS-PAGE and transferred to a nitrocellulose membrane. β-actin was used to ensure equal loading. The results were visualized using a chemiluminescent detection system (Pierce ECL Substrate Western Blot Detection System, Thermo Scientific, IL, USA) and exposure to autoradiography film (Kodak XAR film).

### Statistical analysis

All of the results, with the exception of the results from the Western blot assay, are expressed as the mean values ± standard deviations (SDs) of triplicate independent experiments. *P* values < 0.05 were considered statistically significant.
